# A Predictive Model of 2yDFS During MR-Guided RT Neoadjuvant Chemoradiotherapy in Locally Advanced Rectal Cancer Patients

**DOI:** 10.3389/fonc.2022.831712

**Published:** 2022-02-24

**Authors:** Giuditta Chiloiro, Luca Boldrini, Francesco Preziosi, Davide Cusumano, Poonam Yadav, Angela Romano, Lorenzo Placidi, Jacopo Lenkowicz, Nicola Dinapoli, Michael F. Bassetti, Maria Antonietta Gambacorta, Vincenzo Valentini

**Affiliations:** ^1^ Dipartimento Diagnostica per Immagini, Radioterapia Oncologica ed Ematologia Fondazione Policlinico Universitario Agostino Gemelli Istituto di Ricovero e Cura a Carattere Scientifico (IRCCS), Rome, Italy; ^2^ Dipartimento Universitario di Scienze Radiologiche ed Ematologiche, Università Cattolica del Sacro Cuore, Rome, Italy; ^3^ Department of Human Oncology, School of Medicine and Public Health, University of Wisconsin-Madison, Madison, WI, United States

**Keywords:** delta radiomics, predictive model, rectal cancer, MRgRT, neoadjuvant chemoradiotherapy

## Abstract

**Purpose:**

Distant metastasis is the main cause of treatment failure in locally advanced rectal cancer (LARC) patients, despite the recent improvement in treatment strategies. This study aims to evaluate the “delta radiomics” approach in patients undergoing neoadjuvant chemoradiotherapy (nCRT) treated with 0.35-T magnetic resonance-guided radiotherapy (MRgRT), developing a logistic regression model able to predict 2-year disease-free-survival (2yDFS).

**Methods:**

Patients affected by LARC were enrolled in this multi-institutional study. A predictive model of 2yDFS was developed taking into account both clinical and radiomics variables. Gross tumour volume (GTV) was delineated on the magnetic resonance (MR) images acquired during MRgRT, and 1,067 radiomic features (RF) were extracted using the MODDICOM platform. The performance of RF in predicting 2yDFS was investigated in terms of the Wilcoxon–Mann–Whitney test and area under receiver operating characteristic (ROC) curve (AUC).

**Results:**

48 patients have been retrospectively enrolled, with 8 patients (16.7%) developing distant metastases at the 2-year follow-up. A total of 1,099 variables (1,067 RF and 32 clinical variables) were evaluated in two different models: radiomics and radiomics/clinical. The best-performing 2yDFS predictive model was a delta radiomics one, based on the variation in terms of area/surface ratio between biologically effective doses (BED) at 54 Gy and simulation (AUC of 0.92).

**Conclusions:**

The results of this study suggest a promising role of delta radiomics analysis on 0.35-T MR images in predicting 2yDFS for LARC patients. Further analyses including larger cohorts of patients and an external validation are needed to confirm these preliminary results.

## Introduction

Colorectal cancer (CRC) is one of the most common tumors worldwide and the second most common cause of cancer death in the United States (1).

Rectal cancer represents one-third of all CRCs, with the second highest incidence and second leading cause of cancer death in Western society (2).

Neoadjuvant concurrent chemoradiotherapy (nCRT) or short-course radiotherapy followed by surgery with total mesorectal excision (TME) is considered the standard treatment for locally advanced rectal cancer (LARC) (3, 4).

This treatment approach has led to a significant reduction in local recurrence (LR) (5, 6), although a significant improvement in terms of disease-free survival (DFS) or overall survival (OS) was not demonstrated according to the results of several trials focused on this outcome (7–9).

Despite the improvements in treatment strategies achieved in recent years, distant metastases (DMs) are still the main cause of treatment failure and mortality in LARC patients (10, 11).

Disease-free survival has been used as primary endpoint in numerous trials and represents one of the most promising primary clinical endpoints for patient risk stratification (12).

While 3-year DFS (3yDFS) has been considered a surrogate endpoint for OS in resectable colon cancer, 2-year DFS (2yDFS) has been shown to be a stronger predictor of OS when compared to pathological complete response (pCR) in a pooled analysis of five randomised trials (8). A recent recommendation of outcome measures in rectal cancer suggests 2yDFS as an early predictor of OS and surrogate for good prognosis (12).

Predictive models including clinical data, genetic parameters, and radiomics features extracted from diagnostic images have been proposed for this purpose (13, 14).

More recently, tumor regression measured on magnetic resonance imaging (MRI) before and during nCRT has been applied to predict the pathological response (15, 16).

Fiorino et al. proposed the early regression index (ERI_TCP_) for rectal cancer, a radiobiological parameter based on early regression volume that can predict pCR using 1.5-T staging MR images acquired before and during the neoadjuvant treatment, later confirmed also on 0.35-T hybrid magnetic resonance-guided radiotherapy (MRgRT) images (17, 18).

The introduction of MRgRT indeed opened a new era in radiation oncology, offering the possibility to deliver daily online adaptive treatments and to take benefit of images characterized by higher soft tissue contrast (19, 20).

Besides the blatant advantages related to image quality, the potential role of online MRI as predictors has been investigated in different frameworks (16, 18, 19, 21).

Radiomics and delta radiomics approaches, which study the variations of the radiomics parameters throughout the treatment, have been demonstrated to predict patient response (16, 19).

Furthermore, the change in image-based biomarkers in response to treatment may be used to predict the propensity of the tumour to metastasize, identifying early distant relapse, offering the possibility to fully tailor the follow-up protocols, according to the most innovative paradigms of translational personalized medicine (15, 17).

This multi-institutional retrospective study investigates the delta radiomics approach in patients undergoing nCRT treated with MRgRT, developing a predictive model able to evaluate the 2yDFS probability on hybrid 0.35-T images.

## Materials and Methods

### Study Population

Patients were enrolled from two institutions equipped with 0.35-T MRgRT systems (MRIdian, ViewRay Inc., Cleveland, OH, USA): Fondazione Policlinico Universitario “Agostino Gemelli” IRCCS of Rome, Italy (FPG), and University of Wisconsin-Madison, USA (UW).

All patients were affected by histologically proven locally advanced rectal cancer (cT2–4, cN0–1, cM0), aged ≥18 years, and underwent nCRT on a hybrid 0.35-T MRgRT unit.

Prior to therapy, patients underwent digital rectal examination (DRE), pelvic diagnostic MRI to define T and N staging, contrast-enhanced total-body computed tomography (CT) for M staging, and multidisciplinary tumor board (MDTB) discussion.

Specific informed consent and MRI safety screening forms were administered to all the other patients prior to therapy start.

Patients showing clinical contraindications to MRI or denying specific consent to MRgRT were discarded.

### Treatment Workflow and Treatment Outcome

The FPG patients underwent MRgRT with a simultaneous integrated boost (SIB) technique, delivering 55 Gy (2.2 Gy/fraction) to the gross tumor volume (GTV) and the corresponding mesorectum and 45 Gy (1.8 Gy/fraction) to the mesorectum *in toto* and lymphatic drainage stations, selected according to disease stage (22, 23).

The cohort from UW was treated delivering a sequential treatment, with 50.4 Gy in 28 fractions (1.8 Gy/fraction) to the GTV and corresponding mesorectum and 45 Gy in 25 fractions (1.8 Gy/fraction) to the mesorectum *in toto* and elective lymph node stations, according to institutional guidelines.

Concomitant chemotherapy schedules consisting of oral chronomodulate capecitabine (1,650 mg/mq)/5-fluorouracil (5-FU) 225 mg/mq in continuous infusion or an intensification schedule with capecitabine (1,300 mg/mq) plus oxaliplatin (60 mg/mq) were prescribed for all the Italian patients in relation to the clinical stage and general condition of individual patients.

All the patients treated in UW received concomitant capecitabine (825 mg/mq twice daily during MRgRT).

Clinical restaging was performed 6 to 8 weeks after the end of nCRT through diagnostic MR imaging, DRE, and endoscopic exam, when clinically indicated.

Clinical complete response (cCR) was defined as the absence of palpable masses at DRE and of any mucosal irregularity at endoscopic examination. As for MRI, the rectal wall should appear normal or show only a thin hypointense thickening, with no suspicious lymph nodes visualized and low signal on b1000 images or low apparent diffusion coefficient (ADC) at the previous tumor site (24, 25).

Approximately 4 weeks after clinical restaging, patients underwent surgery that included lower anterior resection (AR), abdominal-perineal resection (APR), and transanal endoscopic microsurgery (TEM) or transanal minimally invasive surgery (TAMIS) in case of major or complete response.

Selected patients underwent a conservative approach in relation to a clinical complete response and MDTB decision (26, 27).

Pathological staging was carried out according to the pTNM classification (28), and the evaluation of the tumour response to neoadjuvant treatment was performed based on tumor regression grade (TRG) according to Mandard’s classification (29, 30).

Adjuvant chemotherapy was administered in selected patients with clinical high-risk factors (cT4 and ypT3–4, ypN1–2, lymphovascular invasion of the tumor, or TRG = 4).

Clinical and imaging follow-up was carried out for all the patients included in the study, for at least 5 years from surgery.

2yDFS was defined as the absence of distant metastasis or local recurrence within 2 years from the end of nCRT.

### MRI Protocol and Statistical Analysis

0.35-T 175-s 3D MR images were acquired using a true fast imaging with steady state precession (TrueFISP) sequence at the simulation phase and before each treatment fraction of the MRgRT treatment.

Images showed T2*/T1 image contrast, with 1.5 × 1.5 × 1.5-mm^3^ spatial resolution and 1.5-mm slice thickness.

A total of five MR images were considered for each patient for the radiomics analysis.

In order to take into account the different fractionation schemes adopted in the two Institutions, the physical doses were converted into biologically effective doses (BED), considering an α/β ratio of 10 (18, 19).

The analyzed MR images were acquired at simulation and when the following biologically effective dose (BED) levels were reached: 13, 26, 40, 54, and 67 Gy.

MR images were then uploaded on a radiotherapy delineation console (Eclipse, Varian Medical System™, Palo Alto, CA, USA) for GTV segmentation, and GTV was contoured following the International Commission on Radiation Units Report 83 (ICRU 83) guidelines by an experienced radiation oncologist skilled in the treatment of lower gastrointestinal malignancies (31).

Contouring and revision of MRI images were blinded for all clinical data including information regarding the achieved pathologic response.

**Figure 1:**
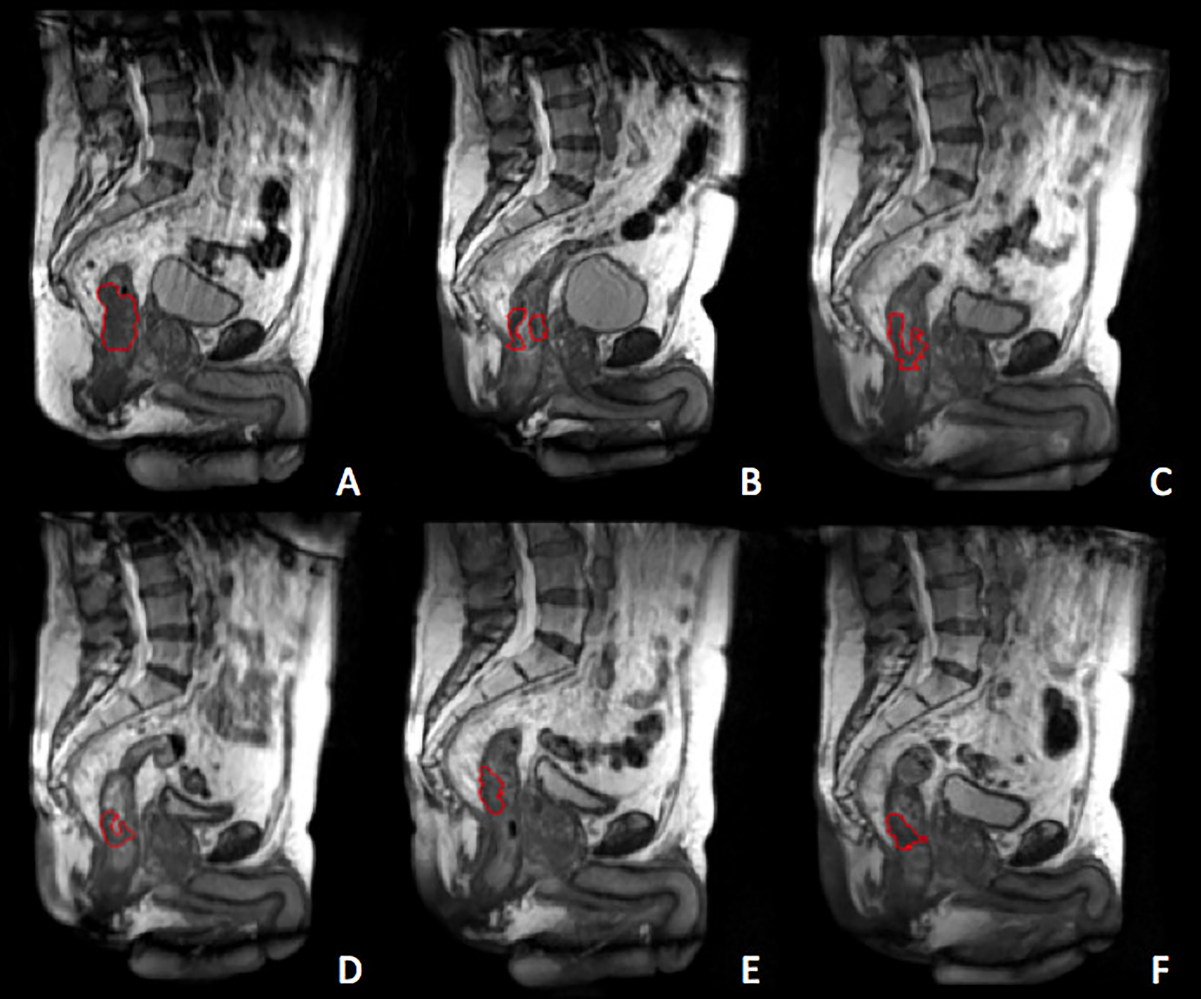
Gross tumour volum (GTV) delineated at the treatment simulation **(A)** and at the different treatment fractions selected for the delta-radiomics analysis, corresponding to BED levels of 13 Gy **(B)**, 26 Gy **(C)**, 40 Gy **(D)**, 54 Gy **(E)** and 67 Gy **(F)**. The GTV is represented by the red contour.

**Figure 1** reports the example of a standard image dataset.

All the imported MR images were processed using signal normalization as described in similar experiences dealing with the same topic (21, 32).

After image preprocessing, radiomics features (RF) belonging to three families (morphological, statistical, textural) were extracted from each MRI considering the GTV as region of interest. Radiomics analysis was performed using MODDICOM, an image biomarker standardization initiative (IBSI) compliant in-house developed radiomics platform (33, 34).

In addition to the standard radiomics features, the variations of RF during treatment were quantified calculating the delta RF, which corresponds to the ratio between the values calculated at different BED levels and the corresponding one extracted at simulation.

A comprehensive database was then created combining the selected radiomics features (RF), general parameters related to diagnosis and treatment (sex, age, clinical TNM staging, radiotherapy dose, type of surgical procedure, and chemotherapy), clinical parameters related to blood analysis (hemoglobin, white blood cells, neutrophils, platelets, neutrophil–lymphocyte ratio), and outcome data (complete or not pathological response, DFS).

Statistical analysis was performed using R software (v 3.4.1, Wien, Austria) and dedicated packages.

The Wilcoxon–Mann–Whitney (WMW) test was performed to identify the ability of each RF in predicting 2yDFS at the univariate analysis.

Pearson correlation coefficient (PCC) was used to estimate the correlation among the delta radiomics features showing statistical significance (p < 0.05) at the univariate analysis.

Two logistic regression linear models were elaborated: the first one using the most significant delta RF, the second one combining two most significant delta RF showing the lowest mutual PCC. The significance performance of such model was evaluated in terms of the receiver operating characteristic curve (ROC) (35).

The area under the ROC curve was considered as the main performance metric, with the 95% confidence intervals calculated using the bootstrap method with 2,000 iterations (36).

The best cutoff threshold was identified maximizing the Youden index, and the values of sensitivity and specificity values at the best threshold were calculated (37).

## Results

A total of 48 consecutive patients were retrospectively enrolled for this analysis: 42 (87.5%) from FPG and 6 (12.5%) from UW.

Overall, the median age for FPG and UW patients was 62 (range 39–87) years and 54 (52–60), respectively. According to the TNM classification, 28 (58.3%) patients presented a cT3 (24 FPG, 4 UW), 13 a cT4 (11 FPG, 2 UW), and 7 a cT2 (7 FPG) disease.

Complete response, considering both patients with persistent cCR and pCR, was reached in 16 cases (33.3%).

Details of patient characteristics and clinical and treatment features are summarized in **Table 1**.

**Table 1:** Patient characteristics.

	Number of patients (%)
**Median age (range)**	62 (39–87)
**Sex**	
**Male**	33 (68.8%)
**Female**	15 (31.2%)
**cT**	
**2**	7 (14.6%)
**3**	28 (58.3%)
**4**	13 (27.1%)
**cN**	
**0**	13 (27.1%)
**+**	35 (72.9%)
**Median radiotherapy dose [Gy] (range)**	55 (50–59.4)
**Median interval between end of nCRT and surgery [weeks] (range)**	14.7 (6–31.4)
**Surgical procedure**	
**APR**	4 (8.3%)
**AR**	32 (66.7%)
**TEM/LE**	2 (4.2%)
**No surgery**	10 (20.8%)
**ypT**	
**0**	8 (21.1%)
**1**	2 (5.3%)
**2**	11 (28.9%)
**3**	17 (44.7%)
**ypN**	
**0**	29 (76.3%)
**+**	9 (23.7%)
**pCR/cCR**	
**Yes**	16 (33.3%)
**No**	32 (66.7%)
**CT type**	
**With Oxa**	17 (35.4%)
**Without Oxa**	2 (4.2%)
**No**	29 (60.4%)
**2yDFS**	
**Yes**	40 (83.3%)
**No**	8 (16.7%)

CT, chemotherapy; pCR, pathological complete response; cCR, clinical complete response; AR, anterior resection/low anterior resection; APR, abdominal-perineal resection; TEM, transanal endoscopic microsurgery; LE, local excision; 2yDFS, 2 year disease free survival.

At a median follow-up of 31 months (range 4–47), 8 patients (16.7%) developed distant metastases, 5 patients at the lung, and 3 at the liver.

A total of 1,099 variables were calculated and evaluated at the univariate analysis, to quantify their ability in predicting the 2yDFS, more specifically, 1,067 RF (582 basal and 485 delta features) and 32 clinical variables.

The most significant RF in identifying 2yDFS was the variation in terms of the area/surface ratio, between BED at 54 Gy and simulation (AV(d4)), which showed an AUC of 0.92 (0.84–1 as 95% confidence interval).

As for clinical features, the type of concomitant and adjuvant chemotherapy showed statistical significance in predicting 2yDFS (p = 0.04).

When combining AV(d4) with the type of chemotherapy (Chemo+AV(d4)), an AUC value of 0.90 (0.81–0.99) was obtained, with a sensitivity of 1.00 and specificity of 0.875 at the best discriminative threshold.

**Figure 2:**
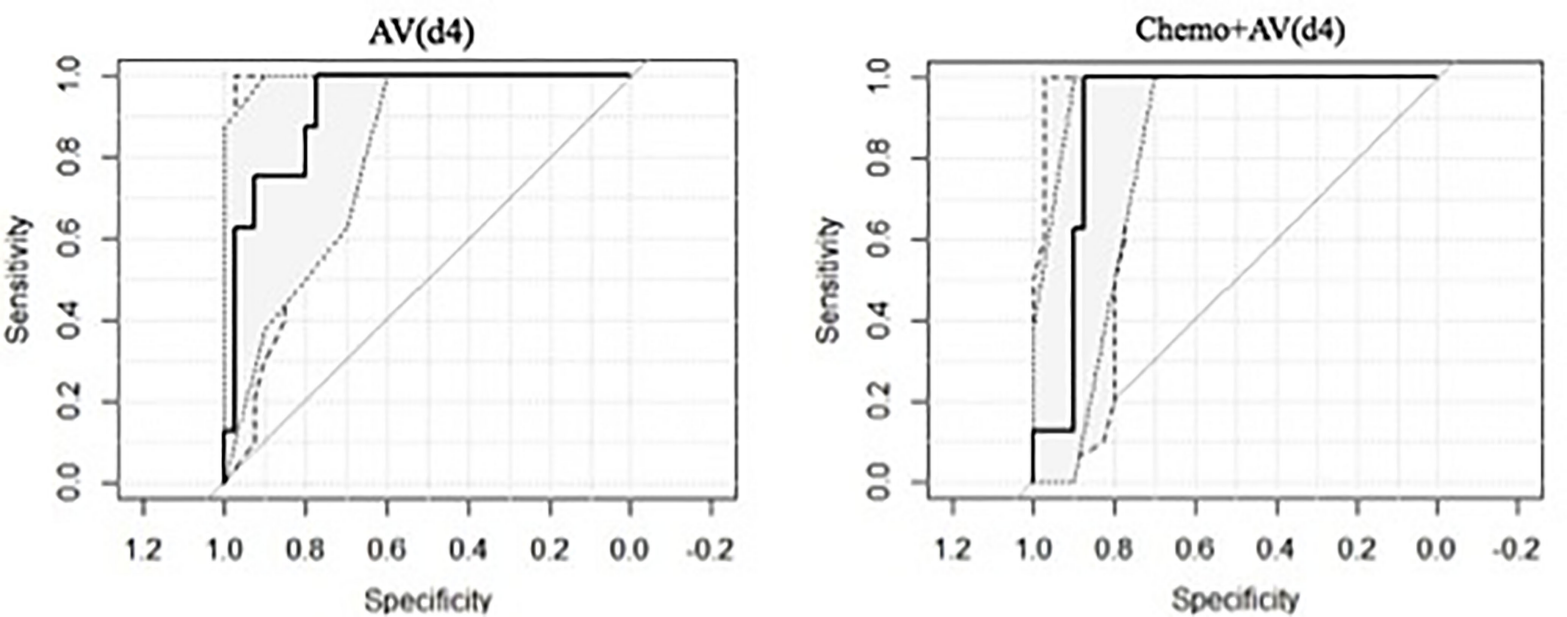
ROC curves of the model elaborated.

The obtained ROC curves are shown in **Figure 2**, while **Table 2** reports values of sensitivity, specificity, and AUC for the two predictive models.

**Table 2:** Sensitivity, specificity, and AUC for the two predictive models.

	Sensitivity	Specificity	Threshold	J_index	AUC	AUC_Low	AUC_High
**Chemo**	0.75	0.700	0.117	-0.985	0.723	0.540	0.906
**AV(d4)**	1.00	0.775	0.138	-0.982	0.925	0.844	1.000
**Chemo+AV(d4)**	1.00	0.875	0.244	-0.981	0.903	0.814	0.991

With regard to the ability of ERI_TCP_ in predicting 2yDFS, the best performance was obtained at a BED level of 40 Gy (p = 0.04), while only limited predictive ability was observed when a BED level of 26 Gy is reached (p = 0.08).

## Discussion

Accurate predictive models are very useful for treatment selection and risk stratification for LARC patients (14), and it is important to define the most appropriate endpoints in order to developed appropriately tailored treatments (12).

DFS is used as outcome in several studies about rectal cancer management (38–40). Basing on the previous work by Valentini et al., 2yDFS has been shown to be a reliable predictor of OS, even stronger than pCR achievement (8).

The feasibility of 2yDFS prediction using a delta radiomics approach was investigated in this retrospective multicentric study in LARC patients undergoing MRgRT.

Boldrini et al. (16) previously demonstrated that delta radiomics and image feature variation during MRgRT treatment may efficaciously describe LARC behavior in terms of post nCRT pCR prediction.

The results here obtained show a 2yDFS predictive ability with an AUC value of 0.92, using the delta AV at the fourth week of treatment.

Interestingly, the combination of delta RF with clinical features (i.e., chemotherapy administration) did not allow to achieve better predictive performances for 2yDFS in LARC patients.

This is in line with the recent studies that evaluated the correlation of delta radiomics features before and after chemoradiotherapy, demonstrating that delta radiomics signatures were independent predictors of treatment response (16, 41).

Besides the very limited delta radiomics experiences, few studies have shown that radiomics features can be extracted from MR scans and used to predict early distant recurrence of LARC patients (15, 42, 43).

A delta radiomics approach was used by Chiloiro et al. to predict the occurrence of DM (15), proposing a model with a balanced accuracy, accuracy, specificity, and sensitivity of 0.785, 0.809, 0.857, and 0.714, respectively. Li et al. (42) developed and validated a combined model that incorporated radiomics features and clinical factors, with an AUC of 0.842 and 0.802 for the training set and validation set, respectively, that may aid in individualized prediction of DM.

Moreover, Liu et al. (43) developed and validated an MRI-based radiomics signature for prediction of DM, by stratifying patients who might benefit from adjuvant chemotherapy.

Latest studies have demonstrated the validity of ERI_TCP_ to predict the pCR status and long-term distant metastasis-free survival (17, 18, 44). Fiorino et al. demonstrated that ERI_TCP_ showed high performances in predicting the pathological response (44) and predicts long-term DM-free survival after nCRT for rectal cancer (17).

Cusumano et al. (18) confirmed the validity of ERI_TCP_ as a pCR predictor in the context of low-tesla MRgRT and indicate 25 Gy as the best BED level to perform predictions.

In the current study, the best predictive performance of ERI_TCP_ was obtained when a BED level of nearly 40 Gy is reached (p = 0.04).

As far as the authors know, this study is the first to develop a predictive model of 2yDFS in rectal cancer using a delta radiomics approach with images acquired during the course of 0.35 T MRgRT.

The most significant radiomics features was AV(d4), which measures the variation of the GTV area/volume ratio between the fourth treatment week and the simulation, again indicating how the morphological variation of GTV during treatment can be predictable of 2yDFS, and not only of pCR, as already demonstrated in some studies (16, 18, 19).

Despite its novelty and methodological robustness, this study suffers some limitations.

First is the relatively small sample size based on retrospective analysis, reflecting the very low number of active MRgRT centers treating LARC patients with long-course radiotherapy.

Second is the limited number of other considered outcomes, which can be used to build a robust and accurate hybrid predictive model.

Last is the lack of a dataset containing a significant number of events to be considered as external validation set, which represents a mandatory step to demonstrate the applicability of the model in a cohort of patients from other institutions and moving toward the clinical application.

Despite these limitations, the results of this study support a promising role of delta radiomics analysis on low-field MR images in predicting 2yDFS for LARC patients.

Further analyses including larger cohorts of patients and a more robust external validation are needed to confirm these preliminary results.

One of the future directions of this work once reaching a significant number of patients will be that of carrying out a dedicated analysis on subpopulations treated with different adjuvant chemotherapy schemes, with the aim of obtaining indications on the optimal chemotherapy approach on the basis of radiomics information extracted from MRI, as already demonstrated on similar experiences carried out on 1.5-T MR images (45).

In the future, predictive models of 2yDFS should be designed in order to identify the subset of patients with a higher risk of DM for a specific adjuvant treatment definition and an effective therapy personalization, including the selection of patients that may benefit from intensified follow-up protocols aiming at the optimization of the available national health system resources.

## Data Availability Statement

The original contributions presented in the study are included in the article/supplementary material. Further inquiries can be directed to the corresponding author.

## Ethics Statement

Ethical review and approval were not required for the study on human participants in accordance with the local legislation and institutional requirements. The patients/participants provided their written informed consent to participate in this study. Written informed consent was obtained from the individual(s) for the publication of any potentially identifiable images or data included in this article.

## Author Contributions

GC, LB, FP, DC, and AR participated in developing the concept of this manuscript, imaging segmentation, data analysis, researching and writing, manuscript preparation, and approval of the final manuscript draft. PY, LP, JL, ND, MFB, MAG, and VV participated in supervision. All authors contributed to the article and approved the submitted version.

## Conflict of Interest

The authors declare that the research was conducted in the absence of any commercial or financial relationships that could be construed as a potential conflict of interest.

## Publisher’s Note

All claims expressed in this article are solely those of the authors and do not necessarily represent those of their affiliated organizations, or those of the publisher, the editors and the reviewers. Any product that may be evaluated in this article, or claim that may be made by its manufacturer, is not guaranteed or endorsed by the publisher.

## References

[1] Siegel RL Miller KD Fuchs HE Jemal A. Cancer Statistics, 2021. CA: A Cancer J Clin (2021) 71:7–33. doi: 10.3322/caac.21654 33433946

[2] Ferlay J Colombet M Soerjomataram I Dyba T Randi G Bettio M. Cancer Incidence and Mortality Patterns in Europe: Estimates for 40 Countries and 25 Major Cancers in 2018. Eur J Cancer (2018) 103:356–87. doi: 10.1016/j.ejca.2018.07.005 30100160

[3] Bosset J-F Collette L Calais G Mineur L Maingon P Radosevic-Jelic L. Chemotherapy With Preoperative Radiotherapy in Rectal Cancer. N Engl J Med (2006) 355:1114–23. doi: 10.1056/nejmoa060829 16971718

[4] Sauer R Becker H Hohenberger W Rödel C Wittekind C Fietkau R. Preoperative Versus Postoperative Chemoradiotherapy for Rectal Cancer. N Engl J Med (2004) 351:1731–40. doi: 10.1056/nejmoa040694 15496622

[5] Lutz MP Zalcberg JR Glynne-Jones R Ruers T Ducreux M Arnold D. Second St. Gallen European Organisation for Research and Treatment of Cancer Gastrointestinal Cancer Conference: Consensus Recommendations on Controversial Issues in the Primary Treatment of Rectal Cancer. Eur J Cancer (2016) 63:11–24. doi: 10.1016/j.ejca.2016.04.010 27254838

[6] Sauer R Liersch T Merkel S Fietkau R Hohenberger W Hess C. Preoperative Versus Postoperative Chemoradiotherapy for Locally Advanced Rectal Cancer: Results of the German CAO/ARO/AIO-94 Randomized Phase III Trial After a Median Follow-Up of 11 Years. J Clin Oncol (2012) 30:1926–33. doi: 10.1200/JCO.2011.40.1836 22529255

[7] Valentini V Gambacorta MA Cellini F Aristei C Coco C Barbaro B. The INTERACT Trial: Long-Term Results of a Randomised Trial on Preoperative Capecitabine-Based Radiochemotherapy Intensified by Concomitant Boost or Oxaliplatin, for Ct2 (Distal)–Ct3 Rectal Cancer. Radiother Oncol (2019) 134:110–8. doi: 10.1016/j.radonc.2018.11.023 31005204

[8] Valentini V Van Stiphout RGPM Lammering G Gambacorta MA Barba MC Bebenek M. Selection of Appropriate End-Points (pCR vs 2ydfs) for Tailoring Treatments With Prediction Models in Locally Advanced Rectal Cancer. Radiother Oncol (2015) 114:302–9. doi: 10.1016/j.radonc.2015.02.001 25716096

[9] Van Gijn W Marijnen CAM Nagtegaal ID Kranenbarg EMK Putter H Wiggers T. Preoperative Radiotherapy Combined With Total Mesorectal Excision for Resectable Rectal Cancer: 12-Year Follow-Up of the Multicentre, Randomised Controlled TME Trial. Lancet Oncol (2011) 12:575–82. doi: 10.1016/S1470-2045(11)70097-3 21596621

[10] Sun Y Lin H Lu X Huang Y Xu Z Huang S. A Nomogram to Predict Distant Metastasis After Neoadjuvant Chemoradiotherapy and Radical Surgery in Patients With Locally Advanced Rectal Cancer. J Surg Oncol (2017) 115:462–9. doi: 10.1002/jso.24522 28105657

[11] Wang J Li S Liu Y Zhang C Li H Lai B. Metastatic Patterns and Survival Outcomes in Patients With Stage IV Colon Cancer: A Population-Based Analysis. Cancer Med (2020) 9:361–73. doi: 10.1002/cam4.2673 PMC6943094 31693304

[12] Fokas E Glynne-Jones R Appelt A Beets-Tan R Beets G Haustermans K. Outcome Measures in Multimodal Rectal Cancer Trials. Lancet Oncol (2020) 21:e252–64. doi: 10.1016/S1470-2045(20)30024-3 32359501

[13] Valentini V Van Stiphout RGPM Lammering G Gambacorta MA Barba MC Bebenek M. Nomograms for Predicting Local Recurrence, Distant Metastases, and Overall Survival for Patients With Locally Advanced Rectal Cancer on the Basis of European Randomized Clinical Trials. J Clin Oncol (2011) 29:3163–72. doi: 10.1200/JCO.2010.33.1595 21747092

[14] Ryan JE Warrier SK Lynch AC Ramsay RG Phillips WA Heriot AG. Predicting Pathological Complete Response to Neoadjuvant Chemoradiotherapy in Locally Advanced Rectal Cancer: A Systematic Review. Colorectal Dis (2016) 18:234–46. doi: 10.1111/codi.13207 26531759

[15] Chiloiro G Rodriguez-Carnero P Lenkowicz J Casà C Masciocchi C Boldrini L. Delta Radiomics Can Predict Distant Metastasis in Locally Advanced Rectal Cancer: The Challenge to Personalize the Cure. Front Oncol (2020) 10:595012. doi: 10.3389/fonc.2020.595012 33344243 PMC7744725

[16] Boldrini L Cusumano D Chiloiro G Casà C Masciocchi C Lenkowicz J. Delta Radiomics for Rectal Cancer Response Prediction With Hybrid 0.35 T Magnetic Resonance-Guided Radiotherapy (MRgRT): A Hypothesis-Generating Study for an Innovative Personalized Medicine Approach. Radiol Med (2019) 124:145–53. doi: 10.1007/s11547-018-0951-y PMC6373341 30374650

[17] Fiorino C Passoni P Palmisano A Gumina C Cattaneo GM Broggi S. Accurate Outcome Prediction After Neo-Adjuvant Radio-Chemotherapy for Rectal Cancer Based on a TCP-Based Early Regression Index. Clin Trans Radiat Oncol (2019) 19:12–6. doi: 10.1016/j.ctro.2019.07.001 PMC6617292 31334366

[18] Cusumano D Boldrini L Yadav P Yu G Musurunu B Chiloiro G. External Validation of Early Regression Index (ERITCP) as Predictor of Pathologic Complete Response in Rectal Cancer Using Magnetic Resonance-Guided Radiation Therapy. Int J Radiat Oncol Biol Phys (2020) 108:1347–56. doi: 10.1016/j.ijrobp.2020.07.2323 32758641

[19] Cusumano D Boldrini L Yadav P Yu G Musurunu B Chiloiro G. Delta Radiomics for Rectal Cancer Response Prediction Using Low Field Magnetic Resonance Guided Radiotherapy: An External Validation. Phys Med (2021) 84:186–91. doi: 10.1016/j.ejmp.2021.03.038 33901863

[20] Meng Y Zhang Y Dong D Li C Liang X Zhang C. Novel Radiomic Signature as a Prognostic Biomarker for Locally Advanced Rectal Cancer. J Magn Reson Imaging (2018) 48:605–14. doi: 10.1002/jmri.25968 29437271

[21] Cusumano D Boldrini L Yadav P Casà C Lee SL Romano A. Delta Radiomics Analysis for Local Control Prediction in Pancreatic Cancer Patients Treated Using Magnetic Resonance Guided Radiotherapy. Diagnostics (2021) 11:72. doi: 10.3390/diagnostics11010072 33466307 PMC7824764

[22] Valentini V Gambacorta MA Barbaro B Chiloiro G Coco C Das P. International Consensus Guidelines on Clinical Target Volume Delineation in Rectal Cancer. Radiother Oncol (2016) 120:195–201. doi: 10.1016/j.radonc.2016.07.017 27528121

[23] Chiloiro G Boldrini L Meldolesi E Re A Cellini F Cusumano D. MR-Guided Radiotherapy in Rectal Cancer: First Clinical Experience of an Innovative Technology. Clin Trans Radiat Oncol (2019) 18:80–6. doi: 10.1016/j.ctro.2019.04.006 PMC6630154 31341981

[24] Chiloiro G Meldolesi E Giraffa M Capocchiano ND Barbaro B Coco C. Could the Conservative Approach Be Considered Safe in the Treatment of Locally Advanced Rectal Cancer in Case of a Clinical Near-Complete or Complete Response? A Retrospective Analysis. Clin Trans Radiat Oncol (2021) 28:1–9. doi: 10.1016/j.ctro.2021.02.009 PMC7937531 33732909

[25] Beets-Tan RGH Lambregts DMJ Maas M Bipat S Barbaro B Curvo-Semedo L. Magnetic Resonance Imaging for Clinical Management of Rectal Cancer: Updated Recommendations From the 2016 European Society of Gastrointestinal and Abdominal Radiology (ESGAR) Consensus Meeting. Eur Radiol (2018) 28:1465–75. doi: 10.1007/s00330-017-5026-2 PMC5834554 29043428

[26] Maas M Beets-Tan RGH Lambregts DMJ Lammering G Nelemans PJ Engelen SME. Wait-And-See Policy for Clinical Complete Responders After Chemoradiation for Rectal Cancer. J Clin Oncol (2011) 29:4633–40. doi: 10.1200/JCO.2011.37.7176 22067400

[27] Martin ST Heneghan HM Winter DC. Systematic Review and Meta-Analysis of Outcomes Following Pathological Complete Response to Neoadjuvant Chemoradiotherapy for Rectal Cancer. Br J Surg (2012) 99:918–928. doi: 10.1002/bjs.8702 22362002

[28] Brierley JD Gospodarowicz MK Wittekind C. TNM Classification of Malignant Tumours. NJ: John Wiley & Sons (2017).

[29] Mandard A-M Dalibard F Mandard J-C Marnay J Henry-Amar M Petiot J-F. Pathologic Assessment of Tumor Regression After Preoperative Chemoradiotherapy of Esophageal Carcinoma. Clinicopathologic Correlations. Cancer (1994) 73:2680–6. doi: 10.1002/1097-0142(19940601)73:11<2680::AID-CNCR2820731105>3.0.CO;2-C 8194005

[30] Vecchio FM Valentini V Minsky BD Padula GDA Venkatraman ES Balducci M. The Relationship of Pathologic Tumor Regression Grade (TRG) and Outcomes After Preoperative Therapy in Rectal Cancer. Int J Radiat Oncol Biol Phys (2005) 62:752–60. doi: 10.1016/j.ijrobp.2004.11.017 15936556

[31] Hodapp N. The ICRU Report No. 83: Prescribing, Recording and Reporting Photon-Beam Intensity-Modulated Radiation Therapy (IMRT). Strahlenther Onkol (2012) 188:97–9. doi: 10.1007/s00066-011-0015-x 22234506

[32] Cusumano D Meijer G Lenkowicz J Chiloiro G Boldrini L Masciocchi C. A Field Strength Independent MR Radiomics Model to Predict Pathological Complete Response in Locally Advanced Rectal Cancer. Radiol Med (2021) 126:421–9. doi: 10.1007/s11547-020-01266-z PMC7937600 32833198

[33] Dinapoli N Alitto AR Vallati M Gatta R Autorino R Boldrini L. Moddicom: A Complete and Easily Accessible Library for Prognostic Evaluations Relying on Image Features. Proc Annu Int Conf IEEE Eng Med Biol Soc EMBS (2015) 2015:771–4. doi: 10.1109/EMBC.2015.7318476 26736376

[34] Zwanenburg A Vallières M Abdalah MA Aerts HJWL Andrearczyk V Apte A. The Image Biomarker Standardization Initiative: Standardized Quantitative Radiomics for High-Throughput Image-Based Phenotyping. Radiology (2020) 295:328–38. doi: 10.1148/radiol.2020191145 PMC7193906 32154773

[35] Yaparpalvi R Hong L Mah D Shen J Mutyala S Spierer M. ICRU Reference Dose in an Era of Intensity-Modulated Radiation Therapy Clinical Trials: Correlation With Planning Target Volume Mean Dose and Suitability for Intensity-Modulated Radiation Therapy Dose Prescription. Radiother Oncol (2008) 89:347–52. doi: 10.1093/jicru/ndn018 18762345

[36] International Commissioning on Radiation Units and Measurements. Receiver Operating Characteristic (ROC) Analysis in Medical Imaging. ICRU Rep (2008) 8:1–62. doi: 10.1093/jicru/ndn008

[37] Ruopp MD Perkins NJ Whitcomb BW Schisterman EF. Youden Index and Optimal Cut-Point Estimated From Observations Affected by a Lower Limit of Detection. Biom J (2008) 50:419–30. doi: 10.1002/bimj.200710415 PMC2515362 18435502

[38] Sargent D Shi Q Yothers G Van Cutsem E Cassidy J Saltz L. Two or Three Year Disease-Free Survival (DFS) as a Primary End-Point in Stage III Adjuvant Colon Cancer Trials With Fluoropyrimidines With or Without Oxaliplatin or Irinotecan: Data From 12,676 Patients From MOSAIC, X-ACT, PETACC-3, C-06, C-07 and C89803. Eur J Cancer (2011) 47:990–6. doi: 10.1016/j.ejca.2010.12.015 PMC3073413 21257306

[39] Smith JJ Chow OS Gollub MJ Nash GM Temple LK Weiser MR. Organ Preservation in Rectal Adenocarcinoma: A Phase II Randomized Controlled Trial Evaluating 3-Year Disease-Free Survival in Patients With Locally Advanced Rectal Cancer Treated With Chemoradiation Plus Induction or Consolidation Chemotherapy, and Total. BMC Cancer (2015) 15:767. doi: 10.1186/s12885-015-1632-z 26497495 PMC4619249

[40] Conroy T Bosset JF Etienne PL Rio E François É Mesgouez-Nebout N. Neoadjuvant Chemotherapy With FOLFIRINOX and Preoperative Chemoradiotherapy for Patients With Locally Advanced Rectal Cancer (UNICANCER-PRODIGE 23): A Multicentre, Randomised, Open-Label, Phase 3 Trial. Lancet Oncol (2021) 22:702–15. doi: 10.1016/S1470-2045(21)00079-6 33862000

[41] Wan L Peng W Zou S Ye F Geng Y Ouyang H. MRI-Based Delta-Radiomics Are Predictive of Pathological Complete Response After Neoadjuvant Chemoradiotherapy in Locally Advanced Rectal Cancer. Acad Radiol (2020) 28 (Suppl 1):S95– 104. doi: 10.1016/j.acra.2020.10.026 33189550

[42] Li M Zhu YZ Zhang YC Yue YF Yu HP Song B. Radiomics of Rectal Cancer for Predicting Distant Metastasis and Overall Survival. World J Gastroenterol (2020) 26:5008–21. doi: 10.3748/wjg.v26.i33.5008 PMC7476170 32952346

[43] Liu Z Meng X Zhang H Li Z Liu J Sun K. Predicting Distant Metastasis and Chemotherapy Benefit in Locally Advanced Rectal Cancer. Nat Commun (2020) 11:4308. doi: 10.1038/s41467-020-18162-9 32855399 PMC7452897

[44] Fiorino C Gumina C Passoni P Palmisano A Broggi S Cattaneo GM. A TCP-Based Early Regression Index Predicts the Pathological Response in Neo-Adjuvant Radio-Chemotherapy of Rectal Cancer. Radiother Oncol (2018) 128:564–8. doi: 10.1016/j.radonc.2018.06.019 30196982

[45] Di Dio C Chiloiro G Cusumano D Catucci F Boldrini L Romano A. Fractal-Based Radiomic Approach to Tailor the Chemotherapy Treatment in Rectal Cancer: A Generating Hypothesis Study. Front Oncol (2021) 11:774413. doi: 10.3389/fonc.2021.774413 34956893 PMC8695680

